# Detection of *Pneumocystis jirovecii* and *Toxoplasma gondii* in patients with lung infections by a duplex qPCR assay

**DOI:** 10.1371/journal.pntd.0010025

**Published:** 2021-12-17

**Authors:** Yun Wu, Fei Wang, Chaoyue Wang, Xinming Tang, Xianyong Liu, Shaogang Li, Nicholas R. Waterfield, Wei Wang, Xun Suo, Guowei Yang

**Affiliations:** 1 Beijing Institute of Tropical Medicine, Beijing Friendship Hospital, Capital Medical University, Beijing, China; 2 Key Laboratory of Animal Epidemiology of Ministry of Agriculture, National Animal Protozoa Laboratory, College of Veterinary Medicine, China Agricultural University, Beijing, China; 3 Institute of Animal Sciences, Chinese Academy of Agricultural Sciences, Beijing, China; 4 Warwick Medical School, Warwick University, Coventry, United Kingdom; 5 Department of Respiratory Medicine, Beijing Friendship Hospital, Capital Medical University, Beijing, China; NIH-National Institute for Research in Tuberculosis-ICER, INDIA

## Abstract

Pneumocystis pneumonia (PCP) and pulmonary toxoplasmosis (PT) are caused by *Pneumocystis jirovecii* and *Toxoplasma gondii*. The clinical symptoms and imaging of PCP and PT are indistinguishable. A duplex qPCR was developed to differentiate between these two pathogens. In testing 92 clinical samples to validate the performance of this method for *P*. *jirovecii* detection, it identified 31 positive samples for *P*. *jirovecii* infection, consistent with clinical diagnosis. Among the remainder of the 61 clinical samples with suspected PCP, yet showing as negative by the conventional PCR diagnosis approach, 6 of them proved positive using our new assay. Our new approach also produced similar results in identification of *T*. *gondii* infections, giving a result of 2 positive and 20 negative in clinical samples. An investigation was undertaken on the prevalence of *P*. *jirovecii* and *T*. *gondii* infections using 113 samples from lung infection patients. 9% (10/113) were shown to be positive with infections of *P*. *jirovecii*, 2% with *T*. *gondii* (2/113) and 5% (6/113) were co-infected with both pathogens. Although this duplex qPCR can detect individual *P*. *jirovecii* and *T*. *gondii* infection, and co-infection of both pathogens, further large-scale investigations are needed to validate its performance, especially in *T*. *gondii* detection. Our assay provides a rapid and accurate tool for PCP and PT diagnosis in immunocompromised population and clinical surveillance of these infections in patients with no immune defects.

## Author summary

*Pneumocystis jirovecii* and *Toxoplasma gondii* are opportunistic pathogens that can cause pneumocystis pneumonia (PCP) and pulmonary toxoplasmosis (PT) in immunocompromised patients. Due to the non-specific clinical symptoms and similar imaging of lung pathology, these two deadly diseases are difficult to be clinically differential diagnosed. Early diagnosis of these infections would reduce medical costs, morbidity and mortality. A duplex qPCR method was developed for the detection of both *P*. *jirovecii* and *T*. *gondii* infection simultaneously. This new assay provides a potential application for diagnosis and surveillance of both PCP and PT. Further investigation for the prevalence of *P*. *jirovecii* and *T*. *gondii* infections indicated that *P*. *jirovecii* and *T*. *gondii* involvement in lung infection has been seriously underestimated.

## Introduction

*Pneumocystis jirovecii* (previously called *P*. *carinii*) and *Toxoplasma gondii* are opportunistic pathogens that can cause pneumocystis pneumonia (PCP) and pulmonary toxoplasmosis (PT), respectively, in immunocompromised patients [1,2]. With the success of antiviral therapy in HIV patients, PCP and PT are now being more commonly observed in patients with other immune deficiency rather than AIDS [3,4], such as patients that had received organ transplantation [5–7], the elderly [8] and infants with suppressed or immature immunity [9,10]. *P*. *jirovecii* infection may be involved in colonization previously or transmission by person to person [11]. *T*. *gondii* can infect through ingestion of cysts from raw or badly-cooked meat, oocysts from substrates contaminated with the feces of infected felines and congenital transmission by tachyzoites [12]. However, these infections are easily neglected or misdiagnosed as other diseases due to the non-specific clinical symptoms and manifestations. In addition, the imaging characteristics of PCP and PT are very similar to each other, they are not distinguishable by clinical lung imaging only. Misdiagnosis of PCP or PT would result in the delayed and insufficient treatment, leading to rapid disease progression with high mortality. The mortality rates for PCP are 4% in AIDS patients and 27% for non-AIDS related patients [4]. While for PT, the mortality rate can reach 43.5% for patients with previous allogeneic haematopoietic stem cell transplantation treatment [3]. Immunocompromised patients are often co-infected with both *P*. *jirovecii* and *T*. *gondii*. The lung infections of these two pathogens are usually manifested mainly as an uncontrolled pneumonia with life threatening outcomes [13–16]. As early diagnosis can improve the clinical outcome, a rapid, accurate and robust tool for *P*. *jirovecii* and *T*. *gondii* diagnosis is urgently needed in clinical practice.

In addition to the analysis of clinical manifestations, imaging, epidemiology and immunocompromised conditions, the accurate and definitive diagnosis of PCP and PT depends on the identification of pathogens using immunological, molecular-biological or microscopic methods [17,18]. Unfortunately, the existing serological antibody tests show cross-reactions with other pathogens and cannot distinguish between current and historical infections. The 1-3-β- glucan is a serum biomarker for PCP diagnosis, however, it is not specific and the cut-off value for PCP diagnosis is not known, limiting its use in clinical practice [19]. Direct fluorescent antibody assay is a reliable method for *P*. *jirovecii* identification while less sensitivity than molecular methods [20]. The microscopic examination for the presence of cysts of *P*. *jirovecii* and *T*. *gondii* in the sputum or bronchoalveolar lavage fluid (BALF) under microscopy is considered as the gold standard for definitive diagnosis of *P*. *jirovecii* and *T*. *gondii* infections. However, this needs an experienced technician or parasitologist as the *P*. *jirovecii* trophozoites are easily missed under microscope, especially when the infection burden is low [21,22]. Parasite culturing of *T*. *gondii* can increase the sensitivity of identification. However, it is time-consuming to culture *T*. *gondii in vitro*. Furthermore, *P*. *jirovecii* cannot be successfully cultured *in vitro*. In addition, conventional PCR, which use mitochondrial large subunit rRNA (mtLSU) as target gene, is currently used in clinical practice [23]. However, compared to conventional PCR, real-time quantitative PCR (qPCR) is more convenient for clinical applications and its results are more reliable.

qPCR plays an important role in *P*. *jirovecii* and *T*. *gondii* detection, which can not only specifically identify the presence of pathogen DNA but also provide a quantitative measurement of the pathogen load [24]. Previous studies have reported qPCR detection of *P*. *jirovecii* based on the mtLSU, NADH dehydrogenase subunit 1 (NAD1), Cytochrome b (CYTB), and Dihydropteroate synthase (DHPS) genes [25]. Among these targets, mtLSU gene was the first one for PCP molecular diagnosis and is still the most widely used [26–30]. For *T*. *gondii* detection, qPCR assays have also been reported with various targets, such as B1, Rep529, COX1, ITS1 [31–33]. However, these previously reported assays are single target qPCR assays designed for the detection of only one pathogen. In this study we developed a duplex qPCR method, targeting the multicopy mitochondrial small subunit rRNA (mtSSU) gene (37 copies per genome) of *P*. *jirovecii* and the repetitive 529 fragment (Rep-529) (200–300 copies per genome) of *T*. *gondii* for the simultaneous detection of both pathogens in patients with lung infections.

## Materials and methods

### Ethics statement

This project has been approved by the Ethics Committee of Beijing Friendship Hospital (Beijing, China) with approval number of 2020-P2-193-01. All clinical samples investigated in this study were obtained from an existing sample collection. All samples were anonymized.

### Patients and clinical and DNA samples

A total of 227 clinical samples from patients at Beijing Friendship Hospital, Capital Medical University were enrolled in this study from Sep 2019 to Sep 2020 (Table 1). The sputum, BALF and blood specimens were collected from them for the duplex qPCR testing. Clinical diagnostic criteria: PCP diagnosis is based on the analysis of the clinical manifestation, imaging characteristics and conventional PCR which amplifies *P*. *jirovecii* mtLSU gene from sputum and BALF specimens [23]. PT diagnosis is based on the analysis of clinical manifestation, imaging characteristics and positive serological IgM and/or IgG [18].

**Table 1:** Characteristics of samples.

Category	Groups	Age	Gender (M%)	Sample type				Conventional PCR		*T*. *gondii* immunoassay		clinical diagnostic criteria or results	Sputum or BALF culture results
				DNA	Sputum (n)	BALF (n)	Blood (n)	Positive (n)	Negative (n)	IgM (n)	IgG (n)		
**Clinical samples for Validation (n = 114)**	PCP (n = 31)	62 (4 months, 84 years)	68%	-	14*	17	0	31	0	-	-	PCP diagnosis is based on the analysis of the clinical manifestation, imaging characteristics and conventional PCR which amplifies *P*. *jirovecii* mtLSU gene from sputum and BALF specimens [23].	-
	Suspected PCP (n = 61)	63 (4 months, 98years)	66%	-	48*	13	0	0	61	-	-		
	*T*. *gondii* infections (n = 2)	6 and 56 years	100%	-	0	0	2	-	-	1	1	PT diagnosis is based on the analysis of clinical manifestation, imaging characteristics and positive serological IgM and/or IgG [18]. Due to lack of commercial kits of qPCR, pathogenic diagnosis relies on immunological methods.	
	Non*-T*. *gondii* infections *(n = 20)*	34 (1, 73) year	70%	-	0	0	20	-	-	0	0		
**Clinical samples for Survey (n = 113)**	Infant (n = 20)	4 (0, 10) days	85%	-	20^#^	0	0			-	-	Neonatal pneumonia; Neonatal sepsis; Tetralogy of fallot; Neonatal wet lung; Respiratory infection	Alpha *hemdytic streptococcus*; *Neisseria; Enterobacter cloacae; Klebsiella oxytoca*
	Elder (n = 76)	79 (55, 104) years	61%	-	58*	8	0			-	-	Pneumonia; Lung cancer; Respiratory infections	Alpha *hemdytic streptococcus; Neisseria; Enterobacter cloacae; Monilia; Acinetobacter baumannii; Klebsiella pneumoniae; Candida tropicalis; Corynebacterium; Stenotrophomonas maltophilia; Pseudomonas aeruginosa*
	Other (n = 17)	48 (3,53) years	59%	-	14*	3	0			-	-	Pneumonia; Respiratory infections	Alpha *hemdytic streptococcus; Neisseria*, *EB virus; Candida albicans; Candida smoothing; Monilia; Stenotrophomonas maltophilia*
**DNA samples (n = 8)**	Positive control	-	-	*P*. *jirovecii* mtSSU plasmid; *T*. *gondii* rep-529 plasmid; *T*. *gondii* parasites DNA	-	-	-	-	-	-	-	-	-
	Negative control	-	-	*alpha hemolytic Streptococcus; Neisseria spp; Mycoplasma;Mycobacterium tuberculosis; Klebsiella pneumoniae*	-	-	-	-	-	-	-		

All data are presented as median (ranges), BALF: bronchoalveolar lavage fluid,—present no data

* represent standard sputum

# represent induced sputum

The 114 samples were used to validate the performance of qPCR assay (Table 1). For *P*. *jirovecii* detection, 31 specimens (14 sputum and 17 BALF) from clinically diagnosed PCP patients confirmed by conventional PCR and 61 samples (48 sputum and 13 BALF) from clinically suspected PCP patients but negative in conventional PCR. For *T*. *gondii* detection, 2 blood samples from patients clinically confirmed with *T*. *gondii* infection, and 20 control blood samples collected from patients with defined malaria (2), dengue fever (1), brucellosis (1) and leishmaniasis (1), or non-defined infection, but all negative when tested with *T*. *gondii* ELISA kit.

Further 113 samples (103 sputum and 10 BALF) from patients with lung infections were used to investigate the prevalence rate of *P*. *jirovecii* and *T*. *gondii* infections (Table 1), including 20 infants, 76 elders and 17 others.

Eight DNA samples were applied in this study (Table 1). *P*. *jirovecii* mtSSU/pUC19 plasmid, *T*. *gondii* rep-529/pUC19 plasmid and DNA samples of *T*. *gondii* parasites are used as positive controls. Negative controls include DNA from *Mycobacterium tuberculosis*, alpha *hemolytic Streptococcus*, *Neisseria*, *Mycoplasma* and *Klebsiella pneumoniae*. In addition, one standard sputum was used for analytical sensitivity analysis.

### Primers and probes design and construction of standard plasmids

Fourteen mtSSU gene sequences from different *P*. *jirovecii* strains and 37 Rep-529 gene sequences from different *T*. *gondii* strains from GenBank were aligned individually using the “Bioedit” software (v7.0.1, Ibis Biosciences, Carlsbad, CA, USA) (S1 and S2 Tables). Primers and probes were designed using Primer Express 3.0 and Oligo 7.0 software based on the conserved region of mtSSU of *P*. *jirovecii* and Rep-529 region of *T*. *gondii* with expected amplification size124bp for *P*. *jirovecii* and 233bp for *T*. *gondii* (Table 2). The specificity of the primers and probes was verified using NCBI Primer blast. The primers and probes were synthesized by Sangon Biotech Co., Ltd.

**Table 2:** Sequence of primers and probes designed for the duplex real-time quantitative PCR.

Species	Target gene	The target gene name	Primer and probe	Sequences	Amplicon size	GeneBank accession No.
*Pneumocystis jirovecii*	mtSSU	Mitochondrial small subunit ribosomal RNA	PJ-F	5’-TTATGAAGTGGGCTACAGAC-3’	124bp	JX499143
			PJ-R	5’-CTTCAAAGAGCCGAGTTCC-3’		
			PJ-probe	5’-FAM-TCCGACTTCCATCATTGCATC-TAMRA-3’		
*Toxoplasma gondii*	Rep-529	529-bp repeat element	TG-F	5’-GACTACAGACGCGATGCC-3’	233bp	DQ779189
			TG-R	5’-CTCTTCAATTCTCTCCGCCAT-3’		
			TG-probe	5’-Texas Red-ACACCGGAATGCGATCTAGACGA-BHQ2-3’		

mtSSU: Mitochondrial small subnuit ribosomal RNA; Rep-529: repetitive 529bp fragment

The mtSSU and Rep-529 target sequence fragments were PCR amplified using the primers described above from the clinical specimens from patients with PCP and cultured *T*. *gondii* parasite respectively, and purified with a DNA purification kit (TIANGEN, DP214, Beijing, CHN), These were then subcloned into plasmid pUC19 (TAKARA, 3219, Tokyo, Japan) using EcoRI and HindIII sites. The correct inserts of target DNA in the recombinant plasmids were confirmed by DNA sequencing.

### DNA extraction

DNA was extracted from fresh sputum or BALF specimens collected from hospitalized patients. The specimens were pre-treated using 1N NaOH (1:1(v/v) for sputum, and 2:1(v/v) for BALF) 60°C for 1h to ensure complete liquefying before centrifugation at 8000 rpm for 5 min. The pellet was washed twice with saline and DNA was extracted from the pellet using a kit (TIANGEN, DP705, Beijing, CHN) according to the manufacturer’s instruction and stored at -80°C for further duplex qPCR assay.

DNA was extracted from blood samples using a DNeasy Blood & Tissue Kit (Qiagen, 69506, Hilden, GER) according to manufacturer’s instructions, which were used as controls for the validation of duplex qPCR assay.

### Duplex quantitative real-time PCR assay

Duplex qPCR was conducted in a 20μl reaction containing 300nm each primers (PJ-F and PJ-R for *P*. *jirovecii*; TG-F and TG-R for *T*. *gondii*) and 200nm each probes (PJ-probe (5′ FAM/3′ TAMRA) for *P*. *jirovecii*; TG-Probe (5′ Texas Red/3′ BHQ2) for *T*. *gondii* with 10μl GoTaq Probe qPCR Master Mix (Promega, A6101, Madison, WI, USA) plus 1μl template DNA (5–50 ng). The reaction was performed in the Applied Biosystems 7500 Fast Real-Time PCR System (ABI) with 95°C for 2 min followed by 40 cycles of 95°C for 15 sec, 58°C for 50 sec. Each sample was tested with replicates, the plasmid mtSSU/pUC19 and Rep-529/pUC19 were used as positive control and reaction without template DNA (distilled water) was used as negative control in all experiments.

### Analytical sensitivity and specificity

The limit of detection (LOD) was determined by minimum number of copies in samples (inter CV%<5%). Briefly, the clinical sample (standard sputum), which was *P*. *jirovecii* and *T*. *gondii* negative, was used as diluents, plasmids mtSSU/pUC19, Rep-529/ pUC19 and mtSSU/pUC19&Rep-529/pUC19 were added in liquefied standard sputum sample and diluted into 1000, 100, 10, 5, 3 or 1 plasmid copies/μl, respectively,the standard sputum sample as blank control. Total DNA were extracted. Detection assay was performed with three batches. The specificity of the duplex qPCR was compared with other DNA samples of obtained *Mycobacterium tuberculosis*, alpha *hemolytic Streptococcus*, *Neisseria* spp, *Mycoplasma* and *Klebsiella pneumoniae*.

### Clinical sensitivity and specificity

Total 114 clinical samples (92 for *P*. *jirovecii* and 22 for *T*. *gondii*) were used to validate the performance of this new assay. DNA extraction and the duplex qPCR was performed according to the procedure described above and the detection results were compared with the clinical diagnosis.

### Prevalence of *P*. *jirovecii* and *T*. *gondii* infections in lung infection patients

The duplex qPCR was further used to test 113 sputum and BALF specimens from lung infection patients (infants n = 20, elders n = 76 and others n = 17) to preliminary investigate the prevalence rate of *P*. *jirovecii* and *T*. *gondii* infections.

### Statistical analysis

Statistical analysis was performed using the SPSS software version 20.0 and visualized on GraphPad Prism version 5.0. Continuous variables were presented as medians (ranges). The Mann-Whitney U-test was used to statistically compare between groups. *P* < 0.05 was considered as statistical significance.

## Results

### Specific detection of *P*. *jirovecii* mtSSU and *T*. *gondii* Rep-529 in a duplex PCR assay

**Fig 1:**
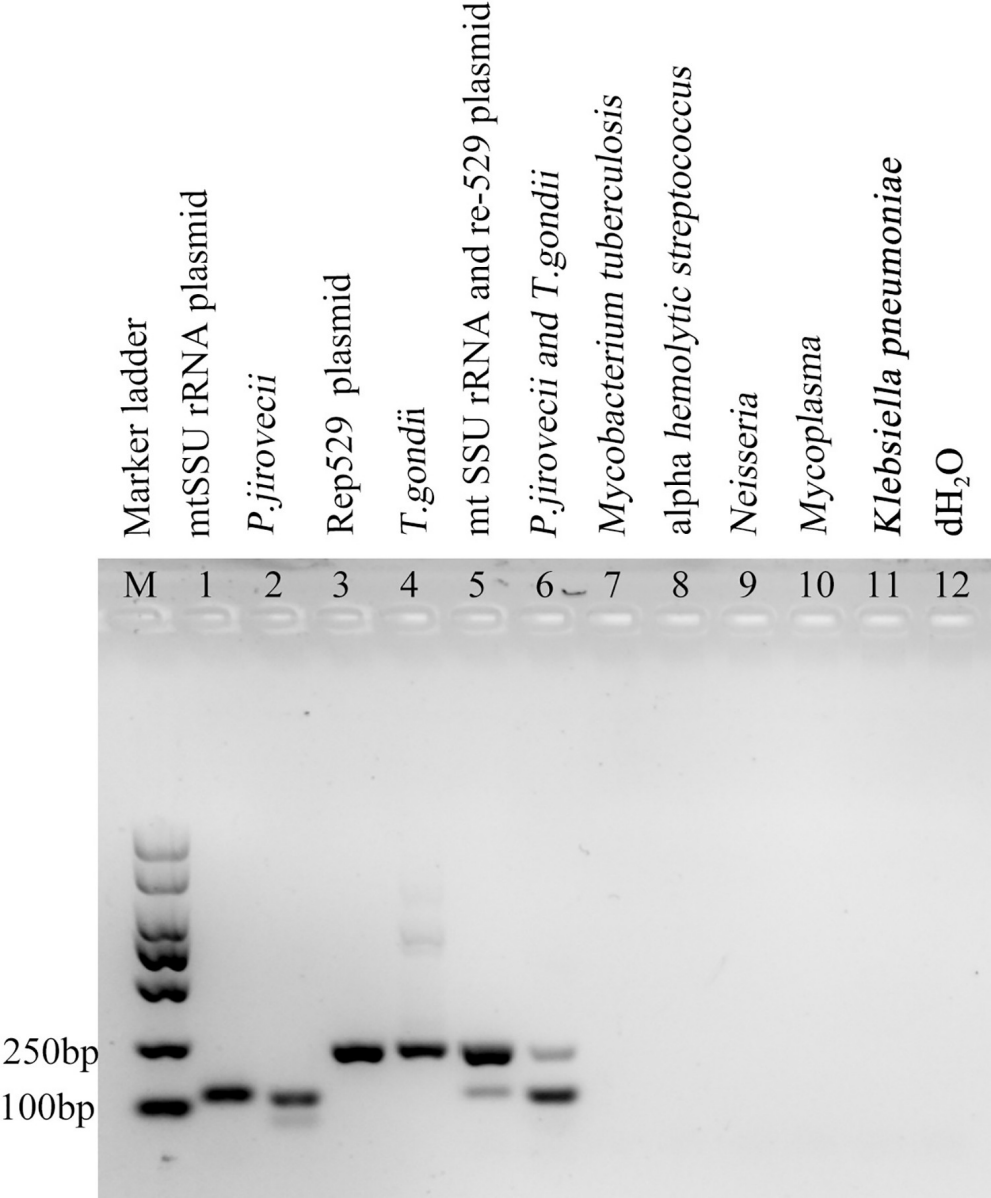
Specificity of the primers designed for *P*. *jirovecii* mtSSU and *T*. *gondii* Rep-529 amplification. The duplex PCR method showed no cross-reaction to *Mycobacterium tuberculosis*, alpha *hemolytic Streptococcus*, *Neisseria*, *Mycoplasma* or *Klebsiella pneumoniae*.

Bioinformatics analysis was firstly performed to locate the potential targets which are conserved among different *P*. *jirovecii* (mtSSU, 99.78% identities) and *T*. *gondii* strains (Rep-529, 92.02% identity) (S1 and S2 Tables). Primers and probes were designed based on these conserved regions (Table 2). Further PCR tests with different DNA samples indicate these primers are highly specific to detect *P*. *jirovecii* and *T*. *gondii* and exhibit no cross-reactions with other pathogens, including *Mycobacterium tuberculosis*, alpha *hemolytic Streptococcus*, *Neisseria*, *Mycoplasma* and *Klebsiella pneumoniae* (Fig 1). Thus, this duplex PCR assay may be suitable to further establish qPCR system to detect both *P*. *jirovecii* and *T*. *gondii* simultaneously.

### Sensitivity of established duplex qPCR assay

**Fig 2:**
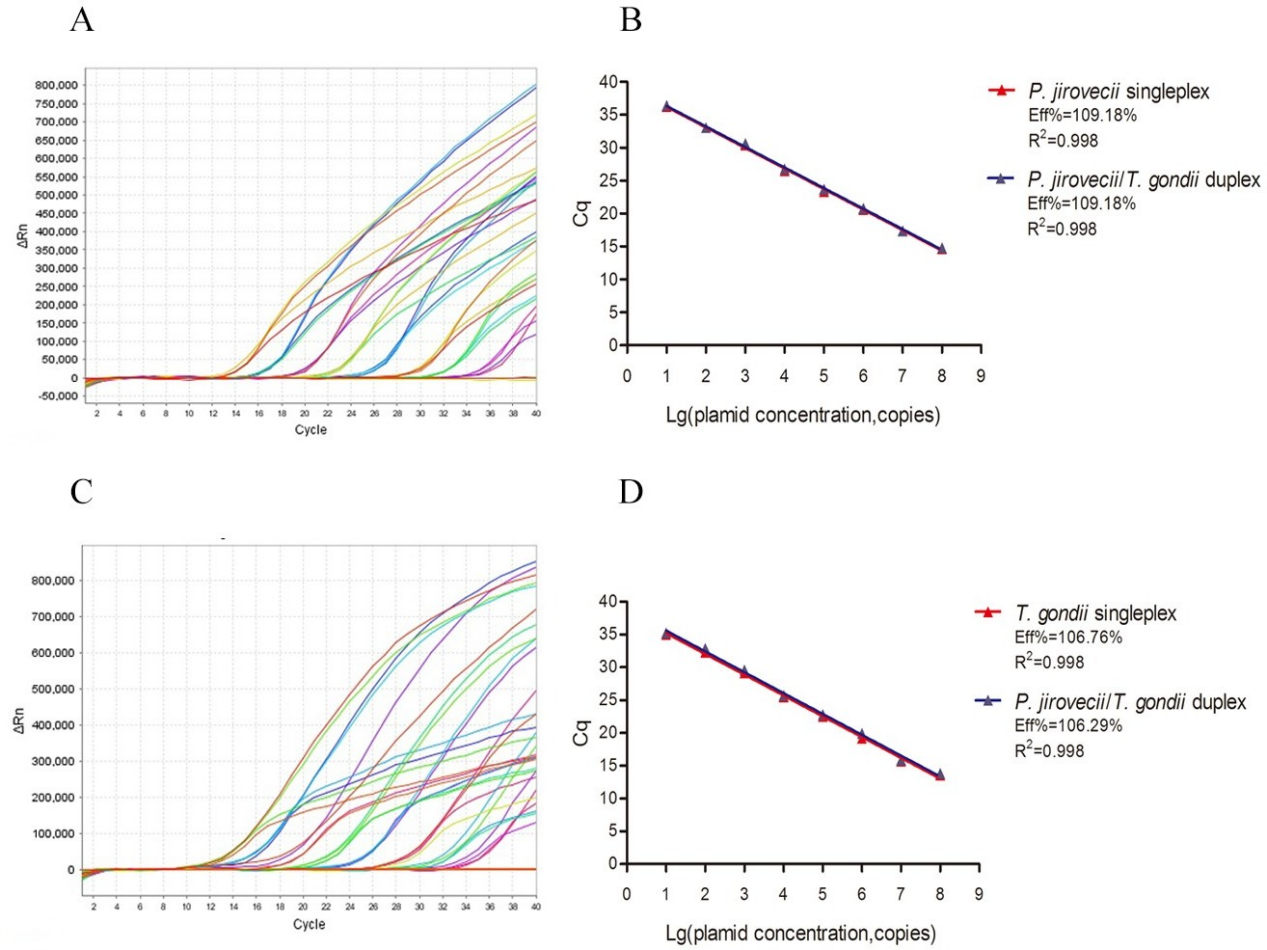
Quantitative correlation between gene copy number and threshold cycle of the duplex qPCR assay. **(A)** *P*. *jirovecii* mtSSU plasmid was serially diluted from 10^1^ to 10^8^ copies/reaction and subjected to qPCR. **(B)** Linear regression of Cq vs. lg copy number of mtSSU plasmid using singleplex qPCR and duplex qPCR. **(C)** *T*. *gondii* Rep-529 plasmid was serially diluted from 10^1^ to 10^8^ copies/reaction and subjected to qPCR. **(D)** Linear regression of Cq vs. lg copy number of Rep-529 plasmid using singleplex qPCR and duplex qPCR. ΔRn = Rn (normalized reporter)-baseline. Ct, Cycle threshold.

A duplex qPCR assay was then established with the primers and probes described above, the LOD of which was 5 copies target DNA for *P*. *jirovecii* and 10 copies for *T*. *gondii* (S3 Table). The standard curves generated by serial copy number dilutions of plasmid DNA using single target qPCR and duplex qPCR, showed that the PCR efficiency in both PCR reactions was the same. The linear range for both were 8-log fold with a correlation coefficient (R^2^) of 0.998 (Fig 2). The intra- assay and inter assay coefficient of variation (CV) of quantification cycle (Cq) values for the 20 replicates was <3%, indicating high precision in the assay (S4 Table). The results indicated that the duplex qPCR assay has high sensitivity and precision to detect target *P*. *jirovecii* mtSSU and *T*. *gondii* Rep-529 genes, ideal for high throughput testing of clinical samples.

### Evaluation of duplex qPCR in the detection of *P*. *jirovecii* and *T*. *gondii* infections in clinical samples

**Fig 3:**
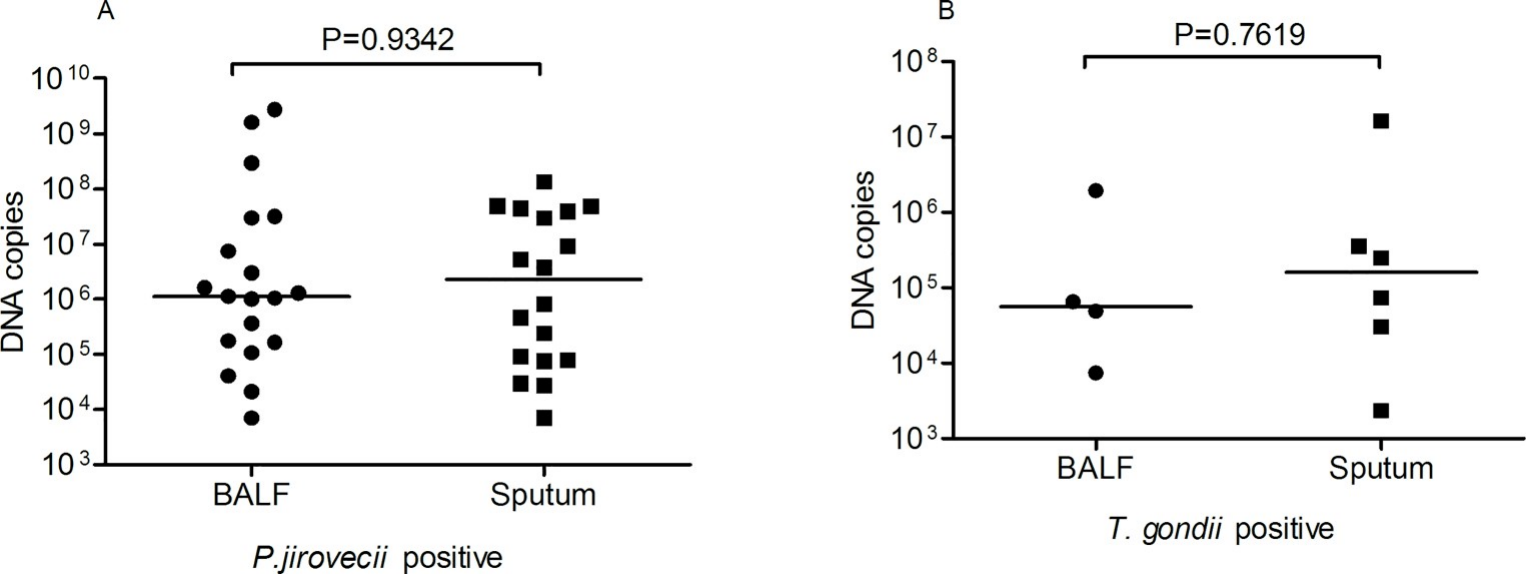
The detected *P*. *jirovecii* (A) and *T*. *gondii* (B) DNA copy number comparison between samples from sputum and BALF.

Total 114 clinical samples were tested to validate the performance of this duplex qPCR assay (Tables 3 and S5). Firstly, the qPCR results revealed that 24 of 31 samples (14 sputum and 17 BALF) from patients with clinically diagnosed PCP were positive for *P*. *jirovecii* only, and 6 were detected as positive for *P*. *jirovecii* only in 61 suspected PCP samples. Secondly, it is interesting to note that 7 of 31 clinically diagnosed PCP samples are co-infected with *T*. *gondii*. Thirdly, 3 of 61 clinically suspected PCP samples were only positive for *T*. *gondii*. Two blood samples with antibodies (IgG/IgM) positive for *T*. *gondii* were positive for *T*. *gondii* DNA even though the detected copy number of Rep-529 gene was low (21 and 190/μl). In addition, twenty control blood samples were all negative for *T*. *gondii*, and all 22 blood samples were negative for *P*. *jirovecii* when the duplex qPCR was performed. The quantitative data showed that there was no significant difference in the amount of DNA between samples from sputum and BALF for *P*. *jirovecii* with DNA copies of 1.1×10^6^ (7×10^3^ to 2.8×10^9^) (Fig 3A). *T*. *gondii* exhibited similar results with DNA copies of 7.03×10^4^ (2.38×10^3^ to 1.64×10^7^) (Fig 3B).

**Table 3:** The duplex qPCR results for 227 clinical samples.

Category	Group	Duplex qPCR							
		*P*. *jirovecii* positive only		*T*. *gondii* positive only		*P*. *jirovecii & T*. *gondii* positive			Negative number
		Number (%)	Cq (ranges)	Number (%)	Cq (range)	Number (%)	*P*. *jirovecii* Cq (ranges)	*T*. *gondii* Cq (ranges)	
**Clinical samples for Validation (n = 114)**	PCP (n = 31)	24/31 (77%)	28.09 (19.24, 36.69)	0	-	7/31 (23%)	30.23 (25.38, 33.19)	32.43 (27.93, 35.61)	0
	Suspected PCP (n = 61)	6/61 (9.83%)	33.31 (27.26, 35.19)	3/61 (5%)	32.61 (24.99, 37.19)	0	-	-	52
	*T*. *gondii* infections (n = 2)	0	-	2	33.99 and 37.23	0	-	-	0
	Non*-T*. *gondii* infections (n = 20)	0	-	0	-	0	-	-	20
	Total (n)	30	-	5	-	7	-	-	72
**Clinical samples for Survey (n = 113)**	Infant (n = 20)	3/20 (15%)	34.55 (33.69, 35.94)	1/20 (5%)	38.28	3/20 (15%)	32.98 (31.03, 35.76)	32.01 (28.06, 33.52)	13
	Elder (n = 76)	7/76 (9%)	33.18 (32.76, 36.87)	1/76 (1%)	29.53	3/76 (4%)	30.94 (28.94, 31.17)	30.21 (29.72, 30.83)	65
	Other (n = 17)	0	-	0	-	0	-	-	17
	Total (n)	10	-	2	-	6	-	-	95

All data are presented as median (ranges), Cq: quantification cycle, n: number

### Prevalence of *P*. *jirovecii* and *T*. *gondii* infections in lung infection patients

**Fig 4:**
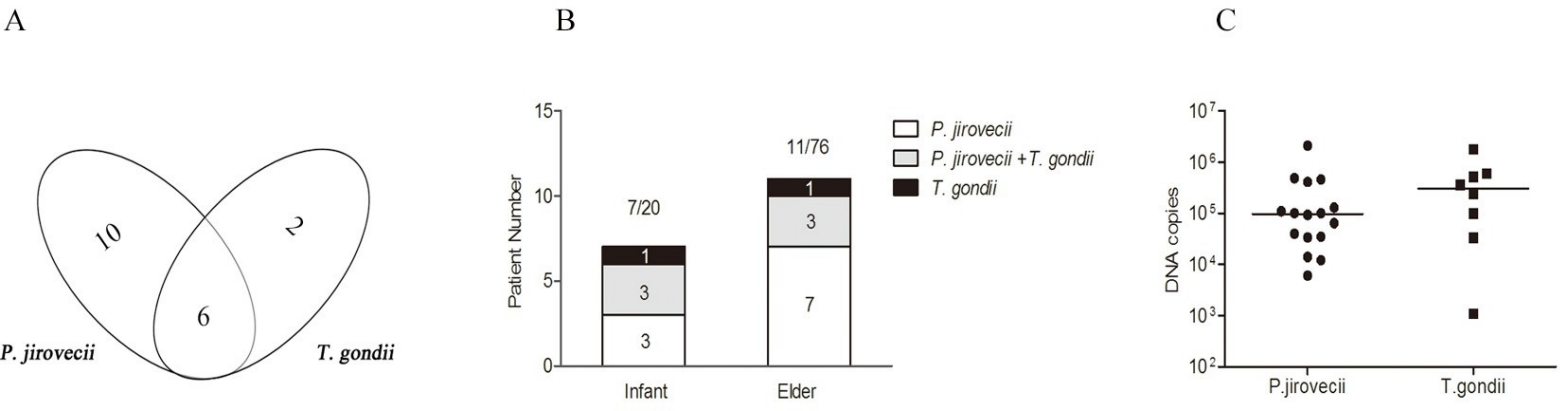
Detection of *P*. *jiroveci*i and *T*. *gondii* infection using duplex qPCR assay on clinical samples from 113 lung infection patients. **(A)** Number of *P*. *jirovecii*, *P*. *jirovecii* + *T*. *gondii* and *T*. *gondii* patients detected by the duplex qPCR. **(B)** *P*. *jirovecii* and *T*. *gondii* positive patients detected with duplex qPCR in infant and elder groups. **(C)** DNA load of *P*. *jirovecii* and *T*. *gondii* detected in lung infection patients.

The duplex qPCR was used to analyze another 113 samples from hospitalized patients with other lung infections (Tables 3 and S5). The results showed that 10 patients were positive for *P*. *jirovecii* only (8.8%), 2 patients were positive for *T*. *gondii* only (1.8%), and 6 patients were positive for both infections (5.3%) (Fig 4A). All infected patients were either the infants with age younger than 4 days or the elder with age over 55 years old. These infected patients include 7 infants with age of 1–4 days old (3 *P*. *jirovecii*, 3 *P*. *jirovecii* co-infection with *T*. *gondii* and 1 *T*. *gondii*) with infection rates of 30% for *P*. *jirovecii* infection (6/20) and 20% for *T*. *gondii* infection (4/20) that are higher than the infection rate in elder patients with age from 55 to 91 years old (7/76 for *P*. *jirovecii*, 1/76 for *T*. *gondii* and 3/76 for co-infection) (Fig 4B). Quantitative results showed that the copy number of *P*. *jirovecii mtSSU* in positive samples (n = 16) was 9.65×10^4^ (6.1×10^3^ to 2.1×10^6^) copies/μl, and the copy number of *T*. *gondii* Rep-529 in positive samples (n = 8) was 3.05×10^5^ (1.1×10^3^ to 1.8×10^6^) copies/μl (Fig 4C).

## Discussion

In our study, we designed a new duplex qPCR based on the conserved regions of the mitochondrial small ribosomal subunit (mtSSU) of *P*. *jirovecii* and the Rep-529 of *T*. *gondii*. The mtSSU rRNA of *P*. *jirovecii* is a multi-copy gene located in the mitochondria of the fungus, with as high as 37 copies in each fungus [25]. This allows for potential higher detection sensitivity compared to other target genes such as mtLSU rRNA (15 copies), NAD1 (23 copies), CYTB (6 copies), and DHPS (single copy). The high copy number of mtSSU rRNA targets in *P*. *jirovecii*, could possibly increase the chance to detect the pathogens even if there is a low infection burden. This target is also highly specific for *P*. *jirovecii* without cross-reaction with other pathogens (Fig 1). For *T*. *gondii*, the Rep-529 loci has been reported to be repeated 200- to 300-fold in the genome of *T*. *gondii* and yielded far higher sensitivity than amplification of B1 sequence (35copies), ITS (110 copies), 18SrDNA (110 copies), SAG (single copy), GRA1 (single copy) genes [25,33–35]. Evaluation of this duplex qPCR assay showed that this method could detect five copies of mtSSU of *P*. *jirovecii* and ten copies of the Rep-529 of *T*. *gondii* from standard sputum samples, and without cross-reaction with other common pathogens causing pneumonia, such as *Mycobacterium tuberculosis*, alpha *hemolytic Streptocoocus*, *Neisseria*, *Mycoplasma*, and *Klebsiella pneumonia*, *Plasmodium*, *Dengue virus*, *Brucella and Leishmania spp*. The high sensitivity and specificity of this duplex qPCR assay suggests that it can be used to diagnose PCP caused by *P*. *jirovecii* infection and PT caused by *T*. *gondii* infection.

To verify this, 92 clinical samples from patients with diagnosed/suspected PCP were used to validate the performance of this assay for *P*. *jirovecii* detection. The results showed that this assay could detect the fungal DNA in all 31 clinically diagnosed PCP samples confirmed by conventional PCR. In 61 samples from patients with suspected PCP based on the PCP clinical manifestation but negative with conventional PCR, 6 of 61 were positive when performed with duplex qPCR, indicating the duplex qPCR has higher sensitivity compared to the standard PCR assay against the *mtLSU* gene [36,37]. As *mtSSU* is 2.5 more copies vs *mtLSU*, it may be the key that this new qPCR assay exhibits higher sensitivity than the current standard method. As for the *T*. *gondii* detection, only two blood samples with clinically confirmed *T*. *gondii* infection (IgM/IgG positive) were used in this study. Considering limited samples size and no sputum or BALF samples were applied in this study, a larger sample size needs to be examined in future to add further confidence for the detection of *T*. *gondii* infections.

This new duplex qPCR method was used to determine the prevalence of *P*. *jirovecii* and *T*. *gondii* infections in 113 lung infection patients. The results showed that 8.8% of them (10/113) were infected with *P*. *jirovecii*, 1.8% with *T*. *gondii* (2/113) and 5.3% (6/113) were co-infected with both *P*. *jirovecii* and *T*. *gondii*. Firstly, this study demonstrates that this designed duplex qPCR can be used to detect not only for individual *P*. *jirovecii* and *T*. *gondii* infections, but also for co-infections in a single assay, with higher sensitivity than the single conventional PCR tests previously reported for individual qPCR assays for these pathogens [38–41]. Secondly, it suggested that infections of *P*. *jirovecii* and *T*. *gondii* are common in patients with general bacterial or virus lung infections, which showed no typical symptoms of pneumonia caused by these two pathogens. It is possibly because they have normal immunity that prevents the pathology caused by these opportunistic pathogens. However, we could not exclude if the lung infection is related to the pathogen infections since there was a lack of detail clinical information. Several studies reported that, colonization rate of *P*. *jirovecii* and *T*. *gondii* reached 37–55% and 19% in patients with lung diseases, respectively [42–44]. Indeed, our results indicated that the copy number of *P*. *jirovecii* DNA from clinical confirmed/suspected samples was significantly higher than that from survey group samples without typical PCP symptoms (P = 0.002) (Figs 3A and 4C). It will be important to determine the correlation between the pathology and the colonized pathogen load, and with the immunological status of the infected people, which needs to examine more specimens in further investigations. Thirdly, some neonates, initially diagnosed as Alpha *hemdytic streptococcus*, *Neisseria* or *Escherichia coli* infections, were also detected positive for *P*. *jirovecii* and/or *T*. *gondii* infections when this duplex qPCR was performed (Table 3). Other studies also reported that, the PCR positive rate of *P*. *jirovecii* and *T*. *gondii* were 15–32% and 9.9%, individually in rhinitis aspirate from hospitalized children (1 month-2 years) with bronchopneumonia or acute respiratory infection and umbilical cord tissue from neonates [45–47]. It indicated that *P*. *jirovecii* and/or *T*. *gondii* infections in neonates were neglected in clinical practice and it should be taken more serious attention in therapeutic management, especially in cases of prematurity, malnutrition and congenital infection. If the congenital toxoplasmosis is suspected or people with infections of *P*. *jirovecii* and *T*. *gondii* is confirmed, the usage of immunosuppressant such as corticoid should be avoided to prevent the diffused infections of these opportunistic pathogens in lungs.

Therefore, our assay provides a rapid and accurate tool for not only diagnosing PCP and PT patients in immunocompromised population, but also clinical surveillance of infections of *P*. *jirovecii* and *T*. *gondii* in patients without immune defects. While the sample size used in this study was relatively small (n = 113), we argue the results are very supportive that this assay is worth pursuing further for the prevalence of *P*. *jirovecii* and *T*. *gondii* infections. A larger cohort of patients should be tested in future to add further confidence in the clinical value of this valuable new diagnostic tool.

In general, *P*. *jirovecii* and *T*. *gondii* involvement in lung infection patients has been seriously underestimated, leading to misdiagnosis and often fatal consequences. Early diagnosis of these infections would reduce medical costs, morbidity and mortality. Our currently designed duplex qPCR detection system in this study exhibits highly sensitivity and specificity to detect *P*. *jirovecii* and *T*. *gondii* infections simultaneously for PCP and PT patients or for populations with no immune defects. It is a fast and simple assay to perform. Further large-scale investigations with more clinical samples should be performed to further evaluate its potential application in clinical practice.

## Supporting information

S1 Table mtSSU Alignment. (XLSX)

S2 Table Rep-529 Alignment. (XLSX)

S3 Table The sensitivity of duplex qPCR. (XLSX)

S4 Table Precision of intra and inter-assay of mtSSU and Rep-529-based qPCR assay. (XLSX)

S5 Table Duplex qPCR assay results. (XLSX)

## References

[1] Alanio A, Hauser PM, Lagrou K, Melchers WJ, Helweg-Larsen J, Matos O, et al. ECIL guidelines for the diagnosis of Pneumocystis jirovecii pneumonia in patients with haematological malignancies and stem cell transplant recipients. J Antimicrob Chemother. 2016;71(9):2386–96. doi: 10.1093/jac/dkw156 . 27550991

[2] Kovari H, Ebnother C, Schweiger A, Berther N, Kuster H, Gunthard HF. Pulmonary toxoplasmosis, a rare but severe manifestation of a common opportunistic infection in late HIV presenters: report of two cases. Infection. 2010;38(2):141–4. doi: 10.1007/s15010-009-9367-5 . 20352286 PMC7102080

[3] Conrad A, Le Marechal M, Dupont D, Ducastelle-Lepretre S, Balsat M, Labussiere-Wallet H, et al. A matched case-control study of toxoplasmosis after allogeneic haematopoietic stem cell transplantation: still a devastating complication. Clin Microbiol Infect. 2016;22(7):636–41. doi: 10.1016/j.cmi.2016.04.025 . 27172809

[4] Roux A, Canet E, Valade S, Gangneux-Robert F, Hamane S, Lafabrie A, et al. Pneumocystis jirovecii pneumonia in patients with or without AIDS, France. Emerg Infect Dis. 2014;20(9):1490–7. doi: 10.3201/eid2009.131668 . 25148074 PMC4178412

[5] Hanisch B, Sprott K, Ardura MI. Pneumocystis jirovecii and toxoplasmosis prophylaxis strategies among pediatric organ transplantation recipients: A US National Survey. Transpl Infect Dis. 2020;22(4):e13290. doi: 10.1111/tid.13290 . 32278336

[6] Robert-Gangneux F, Meroni V, Dupont D, Botterel F, Garcia JMA, Brenier-Pinchart MP, et al. Toxoplasmosis in Transplant Recipients, Europe, 2010–2014. Emerg Infect Dis. 2018;24(8):1497–504. doi: 10.3201/eid2408.180045 . 30014843 PMC6056100

[7] Vindrios W, Argy N, Le Gal S, Lescure FX, Massias L, Le MP, et al. Outbreak of Pneumocystis jirovecii Infection Among Heart Transplant Recipients: Molecular Investigation and Management of an Interhuman Transmission. Clin Infect Dis. 2017;65(7):1120–6. doi: 10.1093/cid/cix495 . 28549105

[8] Abastabar M, Mosayebi E, Shokohi T, Hedayati MT, Jabari Amiri MR, Seifi Z, et al. A multi-centered study of Pneumocystis jirovecii colonization in patients with respiratory disorders: Is there a colonization trend in the elderly? Curr Med Mycol. 2019;5(3):19–25. doi: 10.18502/cmm.5.3.1742 . 31850392 PMC6910707

[9] Lanaspa M, O’Callaghan-Gordo C, Machevo S, Madrid L, Nhampossa T, Acacio S, et al. High prevalence of Pneumocystis jirovecii pneumonia among Mozambican children <5 years of age admitted to hospital with clinical severe pneumonia. Clin Microbiol Infect. 2015;21(11):1018.e9–.e15. doi: 10.1016/j.cmi.2015.07.011 . 26231980

[10] Wang TW, Han YF, Pan ZQ, Wang HZ, Yuan M, Lin H. Seroprevalence of Toxoplasma gondii infection in blood donors in mainland China: a systematic review and meta-analysis. Parasite. 2018;25:36. doi: 10.1051/parasite/2018037 . 30040610 PMC6057739

[11] Morris A, Norris KA. Colonization by Pneumocystis jirovecii and its role in disease. Clin Microbiol Rev. 2012;25(2):297–317. doi: 10.1128/CMR.00013-12 . 22491773 PMC3346301

[12] Lopes FM, Gonçalves DD, Mitsuka-Breganó R, Freire RL, Navarro IT. Toxoplasma gondii infection in pregnancy. The Brazilian journal of infectious diseases: an official publication of the Brazilian Society of Infectious Diseases. 2007;11(5):496–506. doi: 10.1590/s1413-86702007000500011 . 17962877

[13] Ebner L, Walti LN, Rauch A, Furrer H, Cusini A, Meyer AM, et al. Clinical Course, Radiological Manifestations, and Outcome of Pneumocystis jirovecii Pneumonia in HIV Patients and Renal Transplant Recipients. PLoS One. 2016;11(11):e0164320. doi: 10.1371/journal.pone.0164320 . 27824870 PMC5100884

[14] Mayes JT, O’Connor BJ, Robin A, William C, William C. Transmission of Toxoplasma gondii Infection by Liver Transplantation. Clinical Infectious Diseases. 1995;(3):511–5. doi: 10.1093/clinids/21.3.511 . 8527535

[15] Pastorello RG, Costa A, Sawamura MVY, Nicodemo AC, Duarte-Neto AN. Disseminated toxoplasmosis in a patient with advanced acquired immunodeficiency syndrome. Autops Case Rep. 2018;8(1):e2018012. doi: 10.4322/acr.2018.012 . 29588907 PMC5861962

[16] Rottenberg GT, Miszkiel K, Shaw P, Miller RF. Case Report Fulminant Toxoplasma Gondii Pneumonia in a Patient With AIDS. Clinical Radiology. 1997;52:472–4. doi: 10.1016/s0009-9260(97)80012-8 . 9202594

[17] Alanio A, Gits-Muselli M, Guigue N, Desnos-Ollivier M, Calderon EJ, Di Cave D, et al. Diversity of Pneumocystis jirovecii Across Europe: A Multicentre Observational Study. EBioMedicine. 2017;22:155–63. doi: 10.1016/j.ebiom.2017.06.027 . 28705464 PMC5552205

[18] Yan XL, Wen LY, Guan YY, Zhang JF, Lin DD. Iterpretation of criteria for diagnosis of Toxoplasmosis. Chin J Parasitol Parasit Dis. 2016;34(4):387–9. . 30148329

[19] Mercier T, Aissaoui N, Gits-Muselli M, Hamane S, Prattes J, Kessler HH, et al. Variable Correlation between Bronchoalveolar Lavage Fluid Fungal Load and Serum-(1,3)-beta-d-Glucan in Patients with Pneumocystosis-A Multicenter ECMM Excellence Center Study. J Fungi (Basel). 2020;6(4):327. doi: 10.3390/jof6040327 . 33271743 PMC7711754

[20] Donnelly JP, Chen SC, Kauffman CA, Steinbach WJ, Baddley JW, Verweij PE, et al. Revision and Update of the Consensus Definitions of Invasive Fungal Disease From the European Organization for Research and Treatment of Cancer and the Mycoses Study Group Education and Research Consortium. Clin Infect Dis. 2020;71(6):1367–76. doi: 10.1093/cid/ciz1008 . 31802125 PMC7486838

[21] Song YG, Ren Y, Wang XW, Li RY. Recent advances in the diagnosis of pneumocystis pneumonia. Medical Mycology Journal. 2016;57:E111–E6. doi: 10.3314/mmj.16-00019 . 27904052

[22] the Centers for Disease Control and Prevention, the National Institutes of Health, the HIV Medicine Association of the Infectious Diseases Society of America. Guidelines for the prevention and treatment of opportunistic infections in adults and adolescents with HIV. https://clinicalinfohivgov/en/guidelines/adult-and-adolescent-opportunistic-infection/toxoplasma-gondii-encephalitis. updated July 25, 2017; reviewed June 26, 2019.

[23] Wakefield AE, Pixley FJ, Banerji S, Sinclair K, Miller RF, Moxon ER, et al. Detection of Pneumocystis carinii with DNA amplification. The Lancet. 1990;336:451–3. doi: 10.1016/0140-6736(90)92008-6 . 1974987

[24] Desoubeaux G, Dominique M, Morio F, Thepault RA, Franck-Martel C, Tellier AC, et al. Epidemiological Outbreaks of Pneumocystis jirovecii Pneumonia Are Not Limited to Kidney Transplant Recipients: Genotyping Confirms Common Source of Transmission in a Liver Transplantation Unit. J Clin Microbiol. 2016;54(5):1314–20. doi: 10.1128/JCM.00133-16 . 26935726 PMC4844736

[25] Valero C, Buitrago MJ, Gits-Muselli M, Benazra M, Sturny-Leclere A, Hamane S, et al. Copy Number Variation of Mitochondrial DNA Genes in Pneumocystis jirovecii According to the Fungal Load in BAL Specimens. Front Microbiol. 2016;7:1413. doi: 10.3389/fmicb.2016.01413 . 27672381 PMC5018473

[26] Botterel F, Cabaret O, Foulet F, Cordonnier C, Costa JM, Bretagne S. Clinical significance of quantifying Pneumocystis jirovecii DNA by using real-time PCR in bronchoalveolar lavage fluid from immunocompromised patients. J Clin Microbiol. 2012;50(2):227–31. doi: 10.1128/JCM.06036-11 . 22162560 PMC3264196

[27] Guegan H, Robert-Gangneux F. Molecular diagnosis of Pneumocystis pneumonia in immunocompromised patients. Current Opinion in Infectious Diseases. 2019;32(4):314–21. doi: 10.1097/QCO.0000000000000559 . 31107250

[28] Hauser PM, Bille J, Lass-Florl C, Geltner C, Feldmesser M, Levi M, et al. Multicenter, prospective clinical evaluation of respiratory samples from subjects at risk for Pneumocystis jirovecii infection by use of a commercial real-time PCR assay. J Clin Microbiol. 2011;49(5):1872–8. doi: 10.1128/JCM.02390-10 . 21367988 PMC3122670

[29] Edgar RC. MUSCLE: multiple sequence alignment with high accuracy and high throughput. Nucleic Acids Res. 2004;32(5):1792–7. doi: 10.1093/nar/gkh340 . 15034147 PMC390337

[30] Alanio A, Desoubeaux G, Sarfati C, Hamane S, Bergeron A, Azoulay E, et al. Real-time PCR assay-based strategy for differentiation between active Pneumocystis jirovecii pneumonia and colonization in immunocompromised patients. Clin Microbiol Infect. 2011;17(10):1531–7. doi: 10.1111/j.1469-0691.2010.03400.x . 20946413

[31] Ammar NA, Yera H, Bigot J, Botterel F, Hennequin C, Guitard J. Multicentric Evaluation of the Bio-Evolution Toxoplasma gondii Assay for the Detection of Toxoplasma DNA in Immunocompromised Patients. J Clin Microbiol. 2020;58(2):e01231–19. doi: 10.1128/JCM.01231-19 . 31801837 PMC6989062

[32] Gjerde B, Josefsen TD. Molecular characterisation of Sarcocystis lutrae n. sp. and Toxoplasma gondii from the musculature of two Eurasian otters (Lutra lutra) in Norway. Parasitology Research. 2015;114(3):873–86. doi: 10.1007/s00436-014-4251-8 . 25512210

[33] Veronesi F, Santoro A, Milardi GL, Diaferia M, Branciari R, Miraglia D, et al. Comparison of PCR assays targeting the multi-copy targets B1 gene and 529 bp repetitive element for detection of Toxoplasma gondii in swine muscle. Food Microbiology. 2017;63:213–6. doi: 10.1016/j.fm.2016.11.022 . 28040171

[34] Belaz S, Gangneux JP, Dupretz P, Guiguen C, Robert-Gangneux F. A 10-year retrospective comparison of two target sequences, REP-529 and B1, for Toxoplasma gondii detection by quantitative PCR. J Clin Microbiol. 2015;53(4):1294–300. doi: 10.1128/JCM.02900-14 . 25653416 PMC4365238

[35] Homan WL, Vercammen M, Braekeleer RW, Verschueren H. Identification of a 200- to 300-fold repetitive 529 bp DNA fragment in Toxoplasma gondii, and its use for diagnostic and quantitative PCR International Journal for Parasitology. 2000;30 (2000):69–75. doi: 10.1016/s0020-7519(99)00170-8 . 10675747

[36] Gits-Muselli M, White PL, Mengoli C, Chen S, Crowley B, Dingemans G, et al. The Fungal PCR Initiative’s evaluation of in-house and commercial Pneumocystis jirovecii qPCR assays: Toward a standard for a diagnostics assay. Med Mycol. 2020;58(6):779–88. doi: 10.1093/mmy/myz115 . 31758173

[37] Liu BM, Totten M, Nematollahi S, Datta K, Memon W, Marimuthu S, et al. Development and Evaluation of a Fully Automated Molecular Assay Targeting the Mitochondrial Small Subunit rRNA Gene for the Detection of Pneumocystis jirovecii in Bronchoalveolar Lavage Fluid Specimens. The Journal of Molecular Diagnostics. 2020;22(12):1482–93. doi: 10.1016/j.jmoldx.2020.10.003 . 33069878

[38] Ajzenberg D, Lamaury I, Demar M, Vautrin C, Cabie A, Simon S, et al. Performance Testing of PCR Assay in Blood Samples for the Diagnosis of Toxoplasmic Encephalitis in AIDS Patients from the French Departments of America and Genetic Diversity of Toxoplasma gondii: A Prospective and Multicentric Study. PLoS Negl Trop Dis. 2016;10(6):e0004790. doi: 10.1371/journal.pntd.0004790 . 27355620 PMC4927177

[39] Goterris L, Mancebo Fernandez MA, Aguilar-Company J, Falco V, Ruiz-Camps I, Martin-Gomez MT. Molecular Diagnosis of Pneumocystis jirovecii Pneumonia by Use of Oral Wash Samples in Immunocompromised Patients: Usefulness and Importance of the DNA Target. J Clin Microbiol. 2019;57(12):e01287–19. doi: 10.1128/JCM.01287-19 . 31578265 PMC6879283

[40] Khalifa KES, Roth A, Roth B, Arasteh KN, Janitschke K. Value of PCR for Evaluating Occurrence of Parasitemia in immunocompromised patients with cerebral and extracerebral toxoplasmosis. journal of Clinical Microbiology. 1994;32:2813–9. doi: 10.1128/JCM.32.11.2813–2819.1994 . 7852576 PMC264163

[41] Sasso M, Chastang-Dumas E, Bastide S, Alonso S, Lechiche C, Bourgeois N, et al. Performances of Four Real-Time PCR Assays for Diagnosis of Pneumocystis jirovecii Pneumonia. J Clin Microbiol. 2016;54(3):625–30. doi: 10.1128/JCM.02876-15 . 26719435 PMC4767985

[42] Calderon EJ, Rivero L, Respaldiza N, Morilla R, Montes-Cano MA, Friaza V, et al. Systemic inflammation in patients with chronic obstructive pulmonary disease who are colonized with Pneumocystis jiroveci. Clin Infect Dis. 2007;45(2):e17–9. doi: 10.1086/518989 . 17578770

[43] Li YX, Wei CY, Zhang XY, Duan YH, Zhang PN, Guo MJ, et al. Toxoplasma gondii infection in patients with lung diseases in Shandong province, eastern China. Acta Trop. 2020;211:105554. doi: 10.1016/j.actatropica.2020.105554 . 32504591

[44] Wang DD, Zheng MQ, Zhang N, An CL. Investigation of Pneumocystis jirovecii colonization in patients with chronic pulmonary diseases in the People’s Republic of China. Int J Chron Obstruct Pulmon Dis. 2015;10:2079–85. doi: 10.2147/COPD.S89666 . 26491278 PMC4598221

[45] Haq SZ, Abushahama MS, Gerwash O, Hughes JM, Wright EA, Elmahaishi MS, et al. High frequency detection of Toxoplasma gondii DNA in human neonatal tissue from Libya. Trans R Soc Trop Med Hyg. 2016;110(9):551–7. doi: 10.1093/trstmh/trw064 . 27794096

[46] Larsen H, Von Linstow M, Lundgren B, Høgh B, Westh H, Lundgren J. Primary Pneumocystis Infection in Infants Hospitalized with Acute Respiratory Tract Infection. Emerg Infect Dis. 2007;13(1):66–72. doi: 10.3201/eid1301.060315 . 17370517 PMC2725833

[47] Nevez G, Totet A, Pautard JC, Raccurt C. Pneumocystis carinii Detection Using Nested-PCR in Nasopharyngeal Aspirates of Immunocompetent Infants with Bronchiolitis. J Eukaryot Microbiol. 2001:Suppl:122S–3S. doi: 10.1111/j.1550-7408.2001.tb00479.x 11906020

